# A One Health Approach to Address Foodborne Diseases in Low‑ and Middle‑Income Countries

**DOI:** 10.5334/aogh.4708

**Published:** 2025-03-26

**Authors:** Praveen Kumar, Wei Zhang

**Affiliations:** 1 Boston College School of Social Work, Chestnut Hill, USA; 2 International Food Policy Research Institute (IFPRI), Washington, DC, USA

**Keywords:** One health, Foodborne diseases, Food safety

Foodborne diseases (FBDs) are defined as any diseases that result from the ingestion of contaminated or naturally hazardous food [1]. Health outcomes due to FBDs lead to more than 100 million USD of annual preventable economic burden, and over 90% of these economic losses occur in low‑ and middle‑income countries (LMICs) [2]. FBDs disproportionately impact children under 5 years of age. Representing only 9% of the global population, they experience detrimental health outcomes including 38% of all FBD incidence and as much as 30% of premature mortality [3]. Stunting and wasting are pernicious consequences of FBDs among children under 5 years of age. Africa and South‑East Asia have the highest FBD incidence and mortality. Although 41% of the global population are poor, they account for 75% of FBD‑related premature mortality and a 72% loss in global disability‑adjusted life years (DALYs) [3]. In LMICs, FBDs arise primarily owing to the interconnected issues of dwindling animal and plant health, food systems vulnerable to contaminations, and food pathogens and zoonotic threats [1, 2, 4]. There has been a dramatic upsurge in urbanization in LMICs. This trend has accompanied dietary shifts. For example, there is an increase in consumption of animal products (the foods with the highest FBD risk) and intensively managed farming systems. Further, simple food value chains have been supplanted with complex food systems with little traceability. It is now widely acknowledged that human health is intricately linked with animal health, along with our shared ecosystem. The reduction of FBDs, thus, demands a unified framework that pays concurrent attention to the health of humans, animals, and the shared environment. This merits increased attention to adopting a One Health (OH) approach to address FBDs, and by extension, contributing to food security.

## The One Health Framework for Addressing Foodborne Diseases

The Food and Agricultural Organization of the UN (FAO) defines the One Health approach [5] as “an integrated, unifying approach that aims to sustainably balance and optimize the health of people, animals, and ecosystems.” The approach underscores that the health of humans, animals, and ecosystems are closely linked and interdependent. The approach mobilizes multiple sectors, disciplines, and communities at varying levels of society to work together to foster well‑being and tackle threats to health and ecosystems while addressing the collective need for clean water, energy, air, safe and nutritious food, acting on climate change, and contributing to sustainable development. Multiple studies [1, 2, 4, 6] from numerous sources, including the FAO and the World Health Organization (WHO), have emphasized the significance of adopting the OH approach to ensure food safety and reduce FBDs.

## Barriers to the Implementation of the One Health Approach for Reducing FBDs

Two common themes that have emerged from multiple studies on OH and FBDs deserve special mention: (1) knowledge of source attribution, particularly for animal sources as food (ASF); and (2) surveillance of food systems. The clarion call to adopt an OH approach, to enhance source tracing of the food supply chain, and to reduce the incidence and burden of FBDs is commendable. These studies leverage the defining characteristic of the OH approach—cross‑sectoral collaboration to optimize human, animal, and ecosystem well‑being. However, multiple reports fall short of identifying key implementation barriers to realizing the larger goals of the OH approach in food safety. Systematic attention to these implementation challenges is crucial in addressing FBDs with optimal use of resources. Below, we highlight five of these barriers to the implementation of the OH approach in the context of FBDs.

1. Challenges of globalization in food systems: Recent epidemics of swine flu, bird flu, bovine spongiform encephalopathy (BSE), zoonotic influenza, Middle East respiratory syndrome (MERS), and Ebola have raised awareness of adopting the OH approach [7]. The globalization of food supply has created a cross‑border risk for the rapid transfer of food pathogens. The farm‑to‑fork value chain has become complex and challenging to trace [6]. Many LMICs have weak monitoring and database management systems for cross‑border food supply. Poor integration of databases among countries perpetuates this problem. To date, there has been minimal effort in developing a coherent food safety strategy with clear protocols for data collection for all countries to follow and share.
2. Low biosafety levels: Levels of biosafety from animals are generally lower in LMICs owing to factors such as a lack of adequate resources and regulations, and cultural practices [2]. Direct contact between animals (particularly ASF) and humans is more frequent. The likelihood of zoonotic transfer is higher. The OH approach encourages the dissemination of awareness of the hazards of increased human contact with animals. However, low financial support and limited allocation of budgets limit positive outcomes.
3. Perils of demonizing traditional food markets: Despite the growth of supermarkets in middle‑income countries, the majority of ASFs in LMICs are sold in traditional food markets. These are informal commercial hubs, primarily situated in low‑income settings, and have inadequate infrastructure, which increases the risk of food contamination and FBDs [8]. The OH approach advocates for increased surveillance of food supply systems. Most of these traditional food markets, however, are under‑regulated and inadequately monitored. These markets are part of a fragile food system. They are primary sources of food and livelihoods for poor communities in the region. ASFs from supermarkets are largely unaffordable for these poor communities. This leads to a unique situation. Increased surveillance and regulation, in response to the OH approach, may destroy these markets, bringing further misery and nutritional challenges to local poor communities [8]. Instances exist, such as in Zambia, where informal food vendors were relocated to hygienic premises. The relocation limited access to traditional customers and increased transaction costs for vendors [9]. However, continued conditions of lax regulations increase the likelihood of FBDs. Thus, an optimal solution is needed.
4. The dichotomy of stakeholder engagement: The OH approach calls for engaging local and key actors to promote evidence‑based practices to reduce the incidence and burden of FBDs in infrastructure‑constrained settings. Stakeholders’ engagement has mostly led to temporary changes in behavior and perceptions of food safety. Long‑term change merits sustained and inclusive engagement, primarily a function of available resources. In addition, the OH approach promotes long‑term engagement strategies to incorporate local perceptions and belief systems. Educational efforts devoid of local perceptions and belief systems achieve limited buy‑in. However, this creates another challenge: Reliance on local perceptions that are pernicious to food safety perpetuates FBD hazards. For instance, FBDs in a few rural settings in Kenya were seen as indicators of divine punishment [10]. Educational campaigns challenging this notion received lukewarm responses and low vendor engagement [10].
5. Unsustainable agricultural intensification: The last few decades have seen a dramatic increase in agricultural intensification, which accompanies the inordinate application of chemicals, such as fertilizers and pesticides. Evidence shows that intensive agriculture plays a critical role in the rise of antimicrobial resistance (AMR) and dissemination of AMR genes within natural microbial communities [11]. AMR causes drug‑resistant infections in humans that can lead to premature mortality and comorbidities. However, the reduction in (unsustainable) agricultural intensification dents the food supply in LMICs. The existing dilemma deepens the implementation challenges of the OH approach for food safety in LMICs. Measured over the short term (and without accounting for the socio‑environmental externalities including human health outcomes), intensive agriculture still outmatches sustainable agricultural practices in terms of higher food supply with lower input costs.

## Future Action

In 2021, the World Health Organization (WHO) led the formation of a Technical Advisory Group (TAG) on a global food safety strategy for 2022–2023. The TAG underscored the importance of adopting the OH approach to improve food safety by reducing the incidence and prevalence of FBDs. The strategy envisions reducing 40% of FBD diarrheal cases among children under 5 years of age, and targets a universal coordination mechanism to manage FBD events to enhance surveillance. Five strategic priorities were included:

- Strengthening national food control systems
- Identifying and responding to food safety challenges resulting from global changes and food system transformation
- Improving the use of food chain information, scientific evidence, and risk assessment in decisions
- Strengthening stakeholder engagement
- Promoting food safety as an essential component in domestic, regional, and international food trade.

The pathways to realize these strategic priorities are yet to be seen.

The United Nations (UN) defines food insecurity as a lack of adequate, regular, and safe access to nutritious food for normal growth, development, and an active and healthy life. Almost 29.6% of the global population, or 2.4 billion people, were moderately or severely food insecure in 2022 [12]. In total, 11.3% of the global population w severely food insecure [12]. The situation of food insecurity, unfortunately, is more acute for LMICs. Critical regions are Sub‑Saharan Africa, Latin America, and South Asia. Food insecurity has substantially contributed to: (1) malnutrition, and (2) diet‑related non‑communicable diseases (NCDs), as severe public health challenges in LMICs. While obesity is on the rise, malnutrition impacts 1 in 3 children in Africa [13]. An estimated 2–3 billion people cannot afford a healthy diet regularly, which has contributed to diet‑related NCDs, leading to a staggering 73% of premature mortality [14, 15], particularly in LMICs. Population increases, climate change, depletion of natural capital, and political conflicts underpin routine causes of food insecurity. However, foodborne diseases compound food insecurity in LMICs [2, 16], and a deeper understanding of the implementation challenges of the One Health approach is crucial to advance strategies to reduce food insecurity in LMICs. It is crucial to underscore that food security, food safety (or foodborne diseases therein), and human health are tightly interlinked. The realization of their corresponding goals will be co‑dependent on each other.
